# A general pipeline for the development of anchor markers for comparative genomics in plants

**DOI:** 10.1186/1471-2164-7-207

**Published:** 2006-08-14

**Authors:** Jakob Fredslund, Lene H Madsen, Birgit K Hougaard, Anna Marie Nielsen, David Bertioli, Niels Sandal, Jens Stougaard, Leif Schauser

**Affiliations:** 1 Bioinformatics Research Center, University of Aarhus, Høegh-Guldbergs Gade 10, Building 090, DK-8000 Århus C, Denmark; 2 Laboratory of Gene Expression, Department of Molecular Biology, University of Aarhus, Gustav Wieds Vej 10, DK-8000 Århus C, Denmark; 3 Univerisidade Catolica de Brasil – UCB, Programa de Pós-Graduação em Biotecnologia Genômica, Campus II – SGAN Quadra 916, Módulo B, Av. W5 Norte, Brasília – DF, CEP: 70790-160, Brazil

## Abstract

**Background:**

Complete or near-complete genomic sequence information is presently only available for a few plant species representing a large phylogenetic diversity among plants. In order to effectively transfer this information to species lacking sequence information, comparative genomic tools need to be developed. Molecular markers permitting cross-species mapping along co-linear genomic regions are central to comparative genomics. These "anchor" markers, defining unique loci in genetic linkage maps of multiple species, are gene-based and possess a number of features that make them relatively sparse. To identify potential anchor marker sequences more efficiently, we have established an automated bioinformatic pipeline that combines multi-species Expressed Sequence Tags (EST) and genome sequence data.

**Results:**

Taking advantage of sequence data from related species, the pipeline identifies evolutionarily conserved sequences that are likely to define unique orthologous loci in most species of the same phylogenetic clade. The key features are the identification of evolutionarily conserved sequences followed by automated design of intron-flanking Polymerase Chain Reaction (PCR) primer pairs. Polymorphisms can subsequently be identified by size- or sequence variation of PCR products, amplified from mapping parents or populations. We illustrate our procedure in legumes and grasses and exemplify its application in legumes, where model plant studies and the genome- and EST-sequence data available have a potential impact on the breeding of crop species and on our understanding of the evolution of this large and diverse family.

**Conclusion:**

We provide a database of 459 candidate anchor loci which have the potential to serve as map anchors in more than 18,000 legume species, a number of which are of agricultural importance. For grasses, the database contains 1335 candidate anchor loci. Based on this database, we have evaluated 76 candidate anchor loci with respect to marker development in legume species with no sequence information available, demonstrating the validity of this approach.

## Background

The ancestors of crops were originally domesticated and bred because they exhibited specific extraordinary features that made them useful for human consumption, animal food, or ornamental display. Understanding the genetics of these man-selected features involves the identification of underlying qualitative and quantitative trait loci (QTLs) through genetic mapping.

Mapping efficiency and ultimately the ability to isolate interesting loci can be improved by facilitating information transfer from genetic model species to crops and vice versa. Synteny, the conservation of gene order along the chromosomes of related species, is a genomic property that can be exploited for this purpose (reviewed in [1-3]). Shared genetic loci can be used as landmarks for the alignment of linkage groups, thereby defining large chromosomal blocks (macrosynteny). The alignment of sequences from related species often reveals the presence of short regions of sequence conservation (microsynteny). Comparing genomic sequences of genetic models such as Arabidopsis [4] and rice [5,6], with large collections of ESTs from related plants, enables the identification of shared loci instrumental in projecting the large and repetitive genomes of many crop species onto the genomes of the model species. However, comparisons between genetic maps of distantly related species are usually difficult and less productive. Genomes often undergo chromosomal rearrangements, such as inversions, translocations, duplications, deletions and cycles of polyploidization followed by diploidization [7]. Plants, given their sexual promiscuity and potential for vegetative reproduction, are particularly prone to genome rearrangements [8]. For example, whole genome duplications have occurred at several occasions during the evolution of modern plant species [9]. In the diploid phase, members of a duplicated gene pair are retained or deleted at random in the two duplicated regions, obscuring their common past. This process results in diminished congruency between two genomes that are separated by a polyploidization-diploidization cycle. Hence, in order to avoid the pitfalls of comparative genome mapping, the species to be compared should be carefully chosen.

A central step in genome comparisons is the identification of sequences that can readily be identified in genomes of the species to be compared and serve as "anchors" of their respective genetic maps. Commonly used markers, such as microsatellite or AFLP markers, can give high resolution genetic maps, but are of little comparative value since they are not necessarily conserved across species boundaries. Anchor sequences should be chosen to maximize the potential to serve as markers in several species, and allow the quick estimation of congruency between genetic maps of the organisms. First generation comparative maps of plants relied on Southern hybridization of highly conserved homologous probes and their scoring as RFLP markers [10,11]. Markers requiring Southern hybridization, although informative, are time consuming and labor intensive. Furthermore, it can be difficult to generate specific hybridization markers, due to cross-hybridization to other genomic regions. PCR based markers are much more efficient as they are amenable to high throughput automation and, if well designed, of high specificity. Towards this goal we employ a genome-wide strategy based on the identification of low-copy evolutionarily conserved sequences within transcribed sequences of representative species. These regions are used as primer annealing sites for PCR amplification of intercalated introns that are subsequently sequenced in order to ascertain polymorphisms between mapping parents. This approach ensures that non-repetitive, transcribed regions of the genome are the primary targets of mapping efforts.

Here we present an automated pipeline for generation of *C*omparative *A*nchor *T*agged *S*equence markers (CATS [12]) and apply it to design a set of legume anchor markers.

## Results

**Figure 1:**
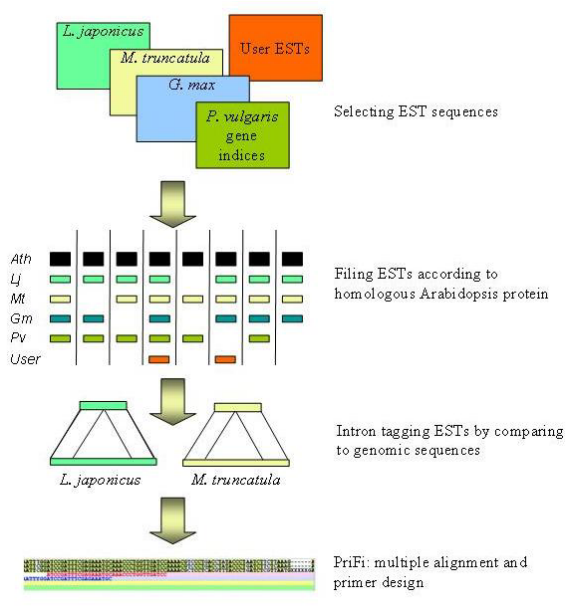
**The pipeline of the marker candidate algorithm**. In the first step, EST collections of selected species are compared with the proteome of the reference species in order to estimate the copy number. Sequences with one or two homologs in the Arabidopsis proteome are considered because Arabidopsis has undergone a recent whole genome duplication whereas legumes have not. EST sequences passing this criterion are compared to *L. japonicus *and *M. truncatula *genomic sequences in order to score the presence and length of introns. Sequences with the same Arabidopsis reference are then aligned and primers are designed using this alignment as input. For this purpose, the PriFi software [13] is used.

The aim of the bioinformatics pipeline was to develop plant family anchor markers useful in order to exploit colinearity between genomes of species with dense genetic maps and crops with important agronomic traits. Sequence polymorphisms constitute the basis of molecular genetics and methods using polymorphisms in noncoding regions like introns are more effective due to differences in evolutionary rate of DNA changes. Functional coding regions and regulatory elements undergo purifying selection, whereas, intron sequences are less constrained and will display a higher degree of mutational variation between any two ecotypes/varieties. Previously, we have developed software for automated multiple alignment-based primer-finding (PriFi) that proposes primer pairs in regions of high conservation for PCR amplification of intervening sequences of low conservation, such as introns [13]. The algorithm employed by the CATS proposing pipeline is best illustrated as a series of consecutive comparative selection filters followed by automated primer-design using PriFi (Figure 1).

### Identification of legume CATS

**Figure 2:**
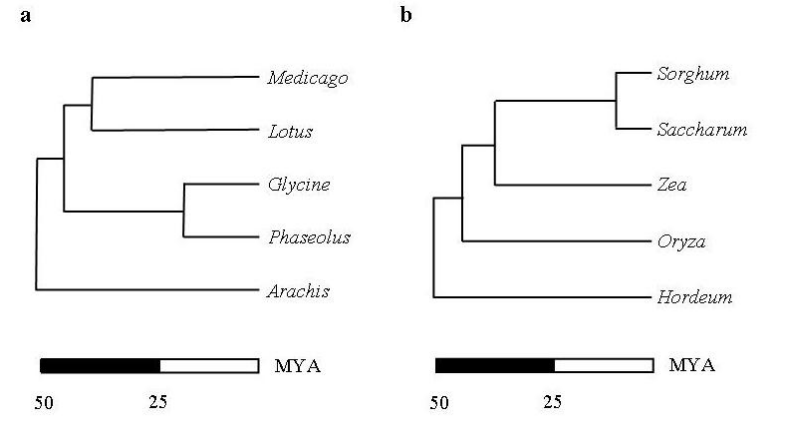
**Phylogenetic trees of legumes and grasses**. Phylogenetic relationship of a) legumes and b) grasses. Species with sequence information used in this study are shown together with selected other species. (Modified after [40])

In the first step of the pipeline the experimenter selects gene sequences for processing. Genome colinearity erodes with phylogenetic distance. It is therefore crucial to choose the resources that allow maximal information transfer between species. Parameters that we considered include the amount of EST information and their phylogenetic relationship. In order to develop legume CATS, we chose resources originating from *Lotus japonicus*, *Medicago truncatula, Phaseolus vulgaris *and *Glycine max*. The phylogenetic relationship between these species is depicted in figure 2a. We have used the ready-clustered EST collections downloadable from TIGR as input, but any assembled collection of ESTs could serve equally well serve as entry points.

Repetitive sequences are not useful for mapping purposes since polymorphisms might reflect paralogous origin rather than allelic variation. Furthermore, allelic variation at a candidate marker locus can be partially or completely masked by the presence of paralogous sequences, reducing the information content of such a marker. For example, the Arabidopsis genome has more than 1000 genes containing the extremely well conserved protein kinase domain. Clearly, such sequences are not well suited to identify unique anchors between genomes. Also, only truly orthologous sequences are able to reveal the syntenic relationships. Homologous single copy genes in any two genomes are very likely being true orthologs. Since no legume genome sequence is complete yet we are relying on legume-derived EST data in this study. The gene copy number in legumes can be indirectly estimated by counting the number of paralogous sequences in the full set of inferred protein-coding sequences (the 'proteome') of a reference species with a complete genome sequence. For legumes, Arabidopsis is the phylogenetically closest species with a complete genome sequence available. Since Arabidopsis has been subject to a recent whole genome duplication [4,16-20], its proteome reference provides a conservative copy number estimate for legume proteomes. Therefore, we allow legume CATS candidates to have one or two homologs in Arabidopsis (Table 1).

**Table 1:** Arabidopsis homolog count for the collections of gene indices.

	*Lotus japonicus *(v. 3.0)	*Medicago truncatula *(v. 8.0)	*Glycine max *(v. 12.0)	*Phaseolus vulgaris *(v. 1.0)
Total number of gene indices	28,460	36,878	63,676	9,484
One Arabidopsis homolog	2,647 (278)	3,613 (869)	4,281	1,441
Two Arabidopsis homologs	1,613 (172)	2,231 (522)	3,349	1,009
Total (one and two Arabidopsis homologs)	4,062 (450)	5,644 (1,391)	7,630	2,450

The species name is followed by the Release version (in parenthesis), and the number of gene indices (GI, combined EST clusters and singleton ESTs) are indicated for each species. Numbers of ESTs with one and two Arabdopsis homologs are listed. The numbers in parenthesis indicate the number of gene indices which show an intron when compared to the respective genomic sequence. *L. japonicus *GIs were compared to *L. japonicus *genomic sequences, whereas *M. truncatula *GIs were compared to *M. truncatula *genomic sequences.

In the next step, the selected EST sequences are aligned with corresponding genomic sequences in order to score intron positions and lengths. This ensures maximal chances of detecting polymorphisms between mapping parents at later steps, since intron sequences are under relaxed evolutionary constraints. Intron position and approximate length, however, are strongly conserved features, even over long evolutionary distances [21]. Here, we make use of the *L. japonicus *and *M. truncatula *genome sequences [23], but the general idea could easily be extended to the Arabidopsis or poplar genome sequences [24], given the conserved nature of intron positions. We also score the length of introns. This parameter is of interest for two reasons: (i) short introns are less likely to be polymorphic than longer ones, and (ii) using standard polymerases the amplicon size is limited to a maximum of ~3 kb. Aligning *L. japonicus *EST sequences to the *L. japonicus *genome identified 450 sequences with introns of appropriate length. Alignment of *M. truncatula *ESTs to the *M. truncatula *genome identified 1,391 sequences with introns of appropriate length.

Next, homologous ESTs from as many as possible of the four legumes with the same best Arabidopsis protein match are aligned. One of the included ESTs must originate from *L. japonicus *or *M. truncatula *and alignment to its gene must reveal the presence of at least one intron of appropriate length.

**Figure 3:**
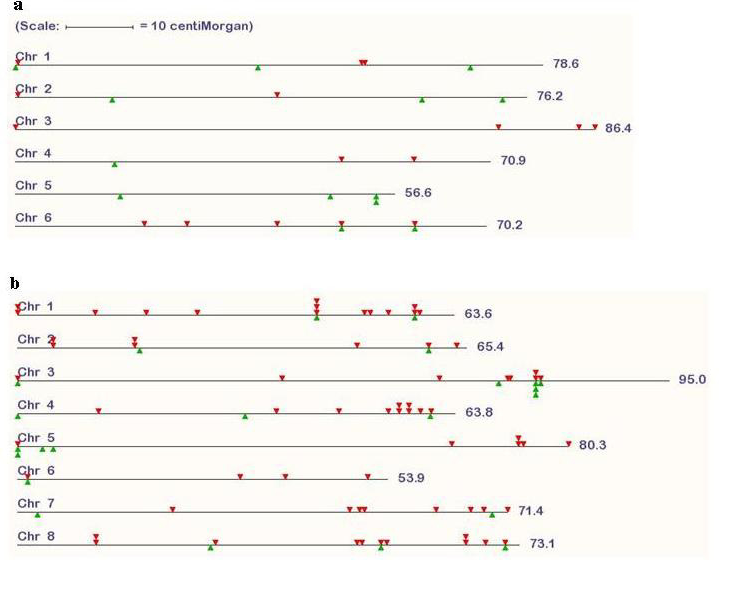
**Distribution of CATS on a) *L. japonicus *b) *M. truncatula *chromosomes**. Red and green triangles indicate positions of markers with one and two homologous gene sequences in Arabidopsis, respectively. Chromosomes scale according to their genetic length.

In a final step, the pipeline designs optimally spaced intron-spanning forward and reverse primers in conserved regions of multiple sequence alignments. To select the optimal primer set among all possible combinations of primers for any multiple alignment, a number of criteria must be met. These include the number of taxons in the alignment, the melting temperature and GC content of the proposed primers and the length of the intron(s) separating the two primers. Also the distance from primer site to the exon-intron junction is considered, since primers that are located too close to an intron will not allow positive sequence confirmation of the PCR product. A combined score for each primer pair allows their comparison and ranking within and between candidate regions. Using stringent cutoff levels, primers for a total of 459 CATS loci were produced applying these steps (Table 2). Of these, we were able to position 29 on the *L. japonicus *genetic map and 66 on the *M. truncatula *map (Figure 3a and 3b). Four markers have map-positions in both maps. Although the sequencing of the two legume genomes is still incomplete, all chromosome arms from both *M. truncatula *and *L. japonicus *are represented.

**Table 2:** The numbers of identified CATS and their anchoring in current *L. japonicus *and *M. truncatula *maps

Reference Genome	*Lotus japonicus*	*Medicago truncatula*
Number of CATS	148	311
Number of map- anchored CATS	29	66

Synteny between to genomic regions can only be established if markers are true orthologs. Orthology between genes from different species is indicated by the matching of gene trees and species tree. To test this, we constructed a Neighbor-joining tree for each alignment. Of a total of 459 gene trees, 57 (12 %) topologies were incompatible with the species tree, potentially due to sequences of paralogous origin in the alignment. These CATS should be treated with care.

The sequence alignments, phylogenetic trees, known map positions, primer reports and gene annotations of all CATS are web-accessible [22].

### Potential for marker development in the grass family

**Figure 4:**
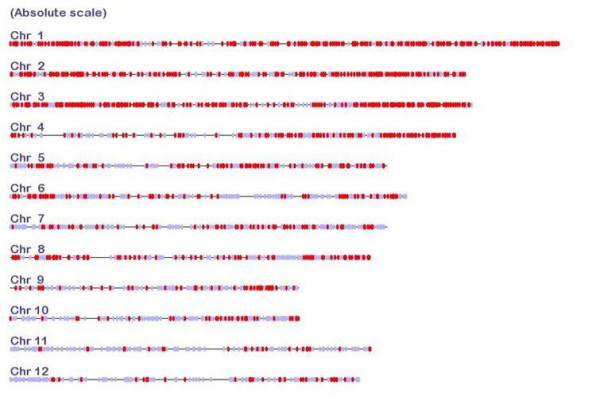
**Distribution of CATS on rice chromosomes**. Red and blue marks indicate the positions of markers with one and two rice homologous gene sequences, respectively. The scale of chromosome diagrams reflects their relative physical sizes.

The development of legume CATS relied on the identification of introns through comparisons of EST sequences to still incomplete *M. truncatula *and *L. japonicus *genomic sequences. Sequencing of both 450 Mbp model legume genomes is still in progress [23], and our analysis is based on 120 Mb of *L. japonicus *genomic sequence and 134 Mb of *M. truncatula *genome sequence. Completion of the gene rich sequences of these genomes will allow more CATS candidates to be identified in the near future. To demonstrate the potential and versatility of the pipeline we applied it to the grass family (Poaceae) where both a complete rice genome and large EST sequence collections are available. The input data files of step one were chosen to represent maximal phylogenetic diversity and sequence information in grasses. The coding sequences of the well annotated rice genome sequence served as one species, and EST collections of wheat and sorghum as the two comparison sequences. Introns were defined by the rice annotation, and the number of paralogous rice sequences was tallied from selfcomparison of the rice proteome. Otherwise we followed the pipeline as outlined for legumes above. In total we were able to identify 1335 grass CATS primer pairs. The distribution of these CATS on the rice chromosomes is shown in Figure 4. All chromosomes are covered. When developed as markers and mapped in several species, these could add considerable density to existing comparative mapping databases such as Gramene [26]. The data for grass CATS developed here is web-accessible [27].

### Testing legume family anchor markers on the outlier peanut

Peanut (*Arachis hypogea*) represents a phylogenetic outgroup to the clade from which the legume sequences were sampled. Hence, it provides a challenging opportunity to assess the potential of our pipeline to generate pan-legume family anchor markers. Recently a microsatellite marker-based genetic map of the AA component of the allotetraploid genome of peanut, has been developed [28]. This map was developed using a mapping population derived from a cross between *A. duranensis*, the most probably AA genome donor of peanut, and the closely related *A. stenosperma*. Anchoring this map to the genome sequence of *L. japonicus *and *M. truncatula *will be a central next step in the development of *Arachis *genetics. Towards this goal, we tested 74 legume family CATS primer pairs which were designed based on the degenerate consensus sequence of the multiple alignment of two to four ESTs included in the legume CATS pipeline. Testing was performed on the in-clade representative common bean and the outlier *Arachis *to evaluate the robustness and general applicability of the pipeline. We determined whether these primer sets amplified the correct sequences, and scored polymorphisms between mapping parents of central mapping populations for both species (see Experimental procedures). Of the 76 tested primer sets, 43 and 56 (or 58 and 75 %) amplified the expected product for *Arachis *and bean, respectively. Sequencing of the PCR products revealed that 79 % of the *Arachis *CATS were polymorphic between *Arachis duranensis *K7988 X *A. stenosperma *V10309. Among the bean CATS, 65 % were polymorphic between the mapping parents Bat93 X JaloEEP558.

## Discussion

The automated bioinformatic pipeline described here allows the large-scale generation of marker candidates useful for map construction and comparisons in legumes and grasses and, by extension, to any phylogenetic clade with appropriate comparative sequence information. Since only unique sequences are unambiguous as markers, the number of paralogous sequences in the target genome is of interest. An approximation to this number is obtained by counting homologous sequences in the proteome of a reference species. Since no legume genome has been completely sequenced to date, the pipeline relies on EST data and is not able to discern between orthologous and paralogous origin of homologous sequences. However, selecting sequences with only one or two homologs in the Arabidopsis reference proteome enhances the probability of an orthologous relationship between homologous legume ESTs. For 88% of the CATS candidate developed here, the gene tree follows the species tree, suggesting orthology. Although these indirect criteria maximize congruency when comparing maps, they by no means guarantee it. A common ancestor of the legumes has undergone a whole-genome duplication [9,16-20], potentially obstructing congruency through differential gene loss in duplicated chromosomal regions. Whereas macrosynteny is not recognizable between *M. truncatula *and Arabidopsis [29], microsynteny is generally much better retained [15,29,30]. Within a given clade, such as legumes or grasses, both micro- and macrosyntenic relations are readily identifiable [8,11,15,31].

A main application of this algorithm is the transfer of genome information between model species and closely related large genome crops. Dense genetic maps spanning all linkage groups are of invaluable help for breeding purposes. Comparison to genomes of other species, especially model species, can help in the development of new markers in regions of interest and may allow educated guesses at candidate genes in the region under investigation. It is therefore advantageous to use markers that can serve as syntenic anchors, connecting genetic maps of as many species as possible.

66 legume CATS were recently published [32]. Here we extend this collection significantly by automating the bioinformatics tasks involved. Applying this pipeline to the currently available legume EST and genome data, we were able to identify 459 CATS. An interactive table of these CATS primers along with EST alignments, BAC sequences and *L. japonicus *and *M. truncatula *map positions is web-available [22].

Currently, the number of CATS to be identified in legumes is limited by the incomplete *M. truncatula *or *L. japonicus *genomic sequences. The sequencing of these genomes is still in progress and will hence allow the identification of many more CATS candidates in the future. Given the conserved nature of intron position and approximate length [21], the Arabidopsis genome sequence might be equally valuable for the identification of introns, a strategy which has been employed by Choi *et al*. [31,32]. However, apart from increasing the number of CATS, such a measure does not allow the linking of genetic map information to sequence information in legumes.

It might be useful to include EST collections from other related species for example ESTs from mung bean (*Vigna mungo*) in the case of the legume family. For this purpose, we have developed a web-based service, GeMprospector [41,42], that allows the user to submit other EST collections to the pipeline, both for legumes and grasses.

The genome sequence used for defining introns makes it possible to sample CATS with even dispersal across all linkage groups or enriched in chromosomal regions of interest. As expected, the success-rate of PCR amplification using degenerate primers decreases with phylogenetic distance of the test species from the species chosen to provide the sequence information for the pipeline. The ability to predict successful primer pairs at the phylogenetic distance between the outlier *Arachis *and the clade defined by *M. truncatua, L. japonicus *and soybean is remarkable and demonstrates the success of the pipeline.

## Conclusion

We present a general framework for the automated design of CATS marker candidates through mining of large-scale sequence collections of diverse origin. The accompanying databases allow legume and grass researchers to get instant and easy access to unprecedented numbers of CATS. Apart from their use as comparative anchor markers, CATS will be useful in the estimation of population genetic parameters such as allele frequencies, effective population sizes and inbreeding coefficients in natural populations and outbreeding crops.

## Methods

### Biological sequence resources

The EST clusters used for this analysis were retrieved from The Institute of Genome Research (TIGR). We downloaded the gene indices (clustered EST collections [34,35]) for the legumes *Lotus japonicus *(Release 3.0: 28,460 sequences), *Medicago truncatula *(Release 8.0: 36,976 sequences), *Phaseolus vulgaris *(Release 1.0: 9,484) and *Glycine max *(Release 12.0: 63,676 sequences) and the grasses *Hordeum vulgare *(Release 9.0: 50,453 sequences), and *Sorghum bicolor*, (Release 8.0: 39,148 sequences). The Arabidopsis and rice (*Oryza sativa*) genome, proteome and coding sequences were downloaded from the TIGR FTP site. The *L. japonicus *and *M. truncatula *genomic sequences were retrieved using NCBIs ENTREZ.

### Bioinformatics resources

The Blast package [36] was obtained from the NCBI. For comparison of nucleotide sequences, we used the Megablast program with a wordsize of 20 and cutoff e-value of 10^-40^. For DNA – protein comparisons, we used the Blastx program (cutoff e-value 2 × 10^-7^). A series of Python scripts were written to parse the Blast outputs and assemble collections of homologous sequences. Multiple alignments were generated using ClustalW [25], and automated primer design was achieved through application of the PriFi program [13]).

CATS were placed on genetic maps of *L. japonicus *[37] and *M. truncatula *[38] by means of the map position of the genomic sequences used for intron finding.

### DNA techniques

DNA was extracted from *Arachis *and bean as described in [28] and [37], respectively. PCR products were sequenced using Applied Biosystems BigDye version 3.1 and run on an ABI PRISM 310 Genetic Analyzer.

The performance of the pipeline was validated experimentally by making use of the suggested primer pairs to amplify homologous sequences from DNA of mapping parents from two legume mapping populations. For bean, the parents of the Bat93 X JaloEEP558 population [39], and for *Arachis *the parents of the *A. duranensis *K7988 X *A. stenosperma *V10309 cross were used [28]. PCR amplification conditions were 40 cycles of [94°C 30 seconds, 48°C 30 seconds, 72°C 2 minutes].

## Authors' contributions

JF wrote all Python, CGI and Html code and conducted all bioinformatics analysis. LHM, BKH, AMN, DB and NS carried out the molecular genetic studies. JS conceived and coordinated the study. LS, JF and LHM designed the study. LS drafted the manuscript. All authors read and approved the final manuscript.

## References

[B1] McCouch SR (2001). Genomics and synteny. Plant Physiol.

[B2] Schmidt R (2000). Synteny: recent advances and future prospects. Curr Opin Plant Biol.

[B3] Delseny M (2004). Re-evaluating the relevance of ancestral shared synteny as a tool for crop improvement. Curr Opin Plant Biol.

[B4] The Arabidopsis Genome Initiative (2000). Analysis of the genome sequence of the flowering plant *Arabidopsis thaliana*. Nature.

[B5] Yu J, Hu S, Wang J, Wong GK, Li S, Liu B, Deng Y, Dai L, Zhou Y, Zhang X, Cao M, Liu J, Sun J, Tang J, Chen Y, Huang X, Lin W, Ye C, Tong W, Cong L, Geng J, Han Y, Li L, Li W, Hu G, Huang X, Li W, Li J, Liu Z, Li L, Liu J, Qi Q, Liu J, Li L, Li T, Wang X, Lu H, Wu T, Zhu M, Ni P, Han H, Dong W, Ren X, Feng X, Cui P, Li X, Wang H, Xu X, Zhai W, Xu Z, Zhang J, He S, Zhang J, Xu J, Zhang K, Zheng X, Dong J, Zeng W, Tao L, Ye J, Tan J, Ren X, Chen X, He J, Liu D, Tian W, Tian C, Xia H, Bao Q, Li G, Gao H, Cao T, Wang J, Zhao W, Li P, Chen W, Wang X, Zhang Y, Hu J, Wang J, Liu S, Yang J, Zhang G, Xiong Y, Li Z, Mao L, Zhou C, Zhu Z, Chen R, Hao B, Zheng W, Chen S, Guo W, Li G, Liu S, Tao M, Wang J, Zhu L, Yuan L, Yang H (2002). A draft sequence of the rice genome (*Oryza sativa *L. ssp. *indica*). Science.

[B6] Goff SA, Ricke D, Lan TH, Presting G, Wang R, Dunn M, Glazebrook J, Sessions A, Oeller P, Varma H, Hadley D, Hutchison D, Martin C, Katagiri F, Lange BM, Moughamer T, Xia Y, Budworth P, Zhong J, Miguel T, Paszkowski U, Zhang S, Colbert M, Sun WL, Chen L, Cooper B, Park S, Wood TC, Mao L, Quail P, Wing R, Dean R, Yu Y, Zharkikh A, Shen R, Sahasrabudhe S, Thomas A, Cannings R, Gutin A, Pruss D, Reid J, Tavtigian S, Mitchell J, Eldredge G, Scholl T, Miller RM, Bhatnagar S, Adey N, Rubano T, Tusneem N, Robinson R, Feldhaus J, Macalma T, Oliphant A, Briggs S (2002). A draft sequence of the rice genome (*Oryza sativa *L. ssp.*japonica*). Science.

[B7] Coghlan A, Eichler EE, Oliver SG, Paterson AH, Stein L (2005). Chromosome evolution in eukaryotes: a multi-kingdom perspective. Trends Genet.

[B8] Bennetzen JL (2000). Comparative sequence analysis of plant nuclear genomes: microcolinearity, its many exceptions. Plant Cell.

[B9] Paterson AH, Bowers JE, Chapman BA (2004). Ancient polyploidization predating divergence of the cereals,, its consequences for comparative genomics. Proc Natl Acad Sci U S A.

[B10] Fulton TM, Van der Hoeven R, Eannetta NT, Tanksley SD (2002). Identification, analysis,, utilization of conserved ortholog set markers for comparative genomics in higher plants. Plant Cell.

[B11] Draye X, Lin YR, Qian XY, Bowers JE, Burow GB, Morrell PL, Peterson DG, Presting GG, Ren SX, Wing RA, Paterson AH (2001). Toward integration of comparative genetic, physical, diversity,, cytomolecular maps for grasses, grains, using the sorghum genome as a foundation. Plant Physiol.

[B12] Lyons LA, Laughlin TF, Copeland NG, Jenkins NA, Womack JE, O'Brien SJ (1997). Comparative anchor tagged sequences (CATS: for integrative mapping of mammalian genomes. Nature Genetics.

[B13] Fredslund J, Schauser L, Madsen LH, Sandal N, Stougaard J (2005). PriFi: using a multiple alignment of related sequences to find primers for amplification of homologs. Nucl Acids Res.

[B14] Schlueter JA, Dixon P, Granger C, Grant D, Clark L, Doyle JJ, Shoemaker RC (2004). Mining EST databases to resolve evolutionary events in major crop species. Genome.

[B15] Mudge J, Cannon SB, Kalo P, Oldroyd GE, Roe BA, Town CD, Young ND (2005). Highly syntenic regions in the genomes of soybean, *Medicago truncatula and Arabidopsis thaliana*. BMC Plant Biol.

[B16] Pfeil BE, Schlueter JA, Shoemaker RC, Doyle JJ (2005). Placing paleopolyploidy in relation to taxon divergence: a phylogenetic analysis in legumes using 39 gene families. Syst Biol.

[B17] Blanc G, Wolfe KH (2004). Widespread paleopolyploidy in model plant species inferred from age distributions of duplicate genes. Plant Cell.

[B18] Vision TJ, Brown DG, Tanksley SD (2000). The origins of genomic duplications in Arabidopsis. Science.

[B19] Simillion C, Vandepoele K, Montagu MC, Zabeau M, Peer Y (2002). The hidden duplication past of *Arabidopsis thaliana*. Proc Natl Acad Sci USA.

[B20] Bowers JE, Chapman BA, Rong J, Paterson AH (2003). Unravelling angiosperm genome evolution by phylogenetic analysis of chromosomal duplication events. Nature.

[B21] Fedorov A, Merican AF, Gilbert W (2002). Large-scale comparison of intron positions among animal, plant,, fungal genes. Proc Natl Acad Sci U S A.

[B22] http://cgi-www.daimi.au.dk/cgi-chili/GeneticMarkers/table.

[B23] Young ND, Cannon SB, Sato S, Kim D, Cook DR, Town CD, Roe BA, Tabata S (2005). Sequencing the genespaces of *Medicago truncatula*, *Lotus japonicus*. Plant Physiol.

[B24] Poplar Genome Consortium. http://genome.jgi-psf.org/Poptr1/Poptr1.home.html.

[B25] Chenna R, Sugawara H, Koike T, Lopez R, Gibson TJ, Higgins DG, Thompson JD (2003). Multiple sequence alignment with the Clustal series of programs. Nucleic Acids Res.

[B26] Ware DH, Jaiswal P, Ni J, Yap IV, Pan X, Clark KY, Teytelman L, Schmidt SC, Zhao W, Chang K, Cartinhour S, Stein LD, McCouch SR (2002). Gramene, a tool for grass genomics. Plant Physiol.

[B27] http://cgi-www.daimi.au.dk/cgi-chili/GeneticMarkers/grass.

[B28] Moretzsohn MC, Leoi L, Proite K, Guimaraes PM, Leal-Bertioli SC, Gimenes MA, Martins WS, Valls JF, Grattapaglia D, Bertioli DJ (2005). A microsatellite-based, gene-rich linkage map for the AA genome of Arachis (Fabaceae). Theor Appl Genet.

[B29] Zhu H, Kim DJ, Baek JM, Choi HK, Ellis LC, Kuester H, McCombie WR, Peng HM, Cook DR (2003). Syntenic relationships between *Medicago truncatula*, Arabidopsis reveal extensive divergence of genome organization. Plant Physiol.

[B30] Krusell L, Madsen LH, Sato S, Aubert G, Genua A, Szczyglowski K, Duc G, Kaneko T, Tabata S, de Bruijn F, Pajuelo E, Sandal N, Stougaard J (2002). Shoot control of root development, nodulation is mediated by a receptor-like kinase. Nature.

[B31] Choi HK, Mun JH, Kim DJ, Zhu H, Baek JM, Mudge J, Roe B, Ellis N, Doyle J, Kiss GB, Young ND, Cook DR (2004). Estimating genome conservation between crop, model legume species. Proc Natl Acad Sci U S A.

[B32] Choi HK, Kim D, Uhm T, Limpens E, Lim H, Mun JH, Kalo P, Penmetsa RV, Seres A, Kulikova O, Roe BA, Bisseling T, Kiss GB, Cook DR (2004). A sequence-based genetic map of *Medicago truncatula *comparison of marker colinearity with *M sativa*. Genetics.

[B33] Zhu H, Choi HK, Cook DR, Shoemaker RC (2005). Bridging model, crop legumes through comparative genomics. Plant Physiol.

[B34] Quackenbush J, Cho J, Lee D, Liang F, Holt I, Karamycheva S, Parvizi B, Pertea G, Sultana R, White J (2001). The TIGR Gene Indices: analysis of gene transcript sequences in highly sampled eukaryotic species. Nucleic Acids Res.

[B35] Pertea G, Huang X, Liang F, Antonescu V, Sultana R, Karamycheva S, Lee Y, White J, Cheung F, Parvizi B, Tsai J, Quackenbush J (2003). TIGR Gene Indices clustering tools (TGICL): a software system for fast clustering of large EST datasets. Bioinformatics.

[B36] Altschul SF, Madden TL, Schaffer AA, Zhang J, Zhang Z, Miller W, Lipman DJ (1997). Gapped BLAST, PSI-BLAST: a new generation of protein database search programs. Nucleic Acids Res.

[B37] Sandal N, Petersen TR, Murray J, Umehara Y, Karas B, Yano K, Kumagai H, Yoshikawa M, Saito K, Hayashi M, Murakami Y, Wang X, Hakoyama T, Imaizumi-Anraku H, Sato S, Kato T, Chen W, Hossain MS, Shibata S, Wang TL, Yokota K, Larsen K, Kanamori N, Madsen E, Radutoiu S, Madsen LH, Radu TG, Krusell L, Ooki Y, Banba M, Betti M, Rispail N, Skot L, Tuck E, Perry J, Yoshida S, Vickers K, Pike J, Mulder L, Charpentier M, Muller J, Ohtomo R, Kojima T, Ando S, Marquez AJ, Gresshoff PM, Harada K, Webb J, Hata S, Suganuma N, Kouchi H, Kawasaki S, Tabata S, Hayashi M, Parniske M, Szczyglowski K, Kawaguchi M, Stougaard J (2006). Genetics of symbiosis in *Lotus japonicus *: recombinant inbred lines, comparative genetic maps, and map position of 35 symbiotic loci. Mol Plant Microbe Interact.

[B38] Medicago truncatula sequencing resources. http://www.medicago.org/genome.

[B39] Freyre R, Skrotch PW, Geffrey V, Adam-Blondon A-F, Shirmohamadali A, Johnson WC, Llaca V, Nodari RO, Pereira PA, Tsai S-M, Tohme J, Dron M, Nienhuis J, Vallejos CE, Gepts P (1998). Towards, integrated map of common bean 4: Development of a core linkage map, alignment of RFLP maps. Theor Appl Genet.

[B40] Doyle JJ, Luckow MA (2003). The rest of the iceberg Legume diversity, evolution in a phylogenetic context. Plant Physiol.

[B41] Fredslund J, Madsen LH, Hougaard BK, Sandal N, Stougaard J, Bertioli D, Schauser L GeMprospector – Online Design of Cross-Species Genetic Marker Candidates in Legumes, Grasses. Nucleic Acids Research.

[B42] http://cgi-www.daimi.au.dk/cgi-chili/GeMprospector/main.

